# Preparation and Assessment of Antimicrobial Effect of Strontium and Copper Co-substituted Hydroxyapatite Nanoparticle-Incorporated Orthodontic Composite: A Preliminary In Vitro Study

**DOI:** 10.7759/cureus.47495

**Published:** 2023-10-22

**Authors:** Raja Kumar, Shweta Nagesh, SP Mani

**Affiliations:** 1 Orthodontics and Dentofacial Orthopaedics, Saveetha Dental College and Hospitals, Saveetha Institute of Medical and Technical Sciences, Saveetha University, Chennai, IND

**Keywords:** nanoparticles, nanohydroxyapatite, orthodontic composite, caries prevention, orthodontics and dentofacial orthopedics

## Abstract

Background and aims

Enamel demineralization and white spot lesions (WSLs) during orthodontic treatment have always been a challenge to orthodontists. The advancement of nanotechnology has paved the way for the incorporation of bioactive compounds in orthodontic materials especially orthodontic composites for prevention and management of WSLs. The present study aims to prepare, characterize, and then incorporate copper and strontium doped nanohydroxyapatite into orthodontic composite material and test its antibacterial efficacy.

Materials and methods

The present in vitro study involved the preparation of the strontium and copper co-substituted hydroxyapatite (SrCuHA) nanoparticles (Nps) using the sol-gel method. The prepared Nps were characterized using scanning electron microscopy (SEM), energy-dispersive X-ray analysis (EDAX), and Fourier transform infrared spectroscopy (FTIR). The Nps were incorporated into a commercially available orthodontic composite. The antimicrobial properties of the SrCuHA Nps-incorporated composite were tested using the Agar well diffusion method against Staphylococcus aureus(*S. aureus*), Streptococcus mutans (*S. mutans*), and Escherichia coli (*E. coli*).

Results

The SrCuHA Nps were successfully prepared. EDAX, FTIR, and SEM analyses revealed the successful formation of the Nps. The SrCuHA-incorporated orthodontic composite at a higher concentration of 40 μl showed the maximum zone of inhibition (ZOI) against *S. mutans*. The control group showed the maximum ZOI against *E. coli *and the SrCuHA Nps-incorporated composite at 20 μl showed the maximum inhibition against *S. aureus*.

Conclusion

In the present study, successful preparation of SrCuHA Nps followed by incorporation in the orthodontic adhesive was done. The prepared nanoparticle was characterized and the SrCuHA Nps-incorporated orthodontic composite demonstrated comparable ZOI against *S. mutans *to the control.

## Introduction

A major problem associated with orthodontic therapy is the decalcification of enamel surfaces around the orthodontic brackets [1]. During orthodontic treatment, the number of oral bacteria increases because brackets, arch wires, and orthodontic accessories can make oral hygiene maintenance difficult [2,3]. Despite many advances, patients undergoing fixed orthodontic treatment remain at a high risk of developing white spot lesions (WSLs). With incidence and prevalence rates of 45.8% and 68.4%, respectively, lesions can start to form as early as four weeks, making the search for prevention methods necessary. WSLs are a major issue for both orthodontists and orthodontic patients since they are unsightly and occasionally irreversible. The traditional means of preventing such lesions include practicing good oral hygiene but patient compliance is a concern [4-6]. Numerous methods have been developed and proposed to reduce WSLs, some of which may include professional fluoride application, fluoride toothpaste, fluoride mousses, and mouthwashes [7]. There has been a significant amount of research on the use of novel materials in the treatment of WSLs [8]. Advances in nanotechnology are paving the way for novel methods to prevent WSLs. Because of their size and surface area, nanoparticles (Nps) have high bactericidal capabilities and are chemically and physiologically active. The mechanical and physical properties of restorative materials and adhesive systems are improved by the use of Nps [9]. There are two basic ways that nanotechnology can combat enamel demineralization. The first technique makes use of nanomaterials with antibacterial capabilities. The second includes using substances that can release calcium and fluoride, like calcium hydroxyapatite (Ca_10_(PO_4_)_6_(OH)_2_) and calcium fluoride (CaF_2_) [10,11].

Hydroxyapatite Nps (HA Nps) has been used extensively in the field of dentistry. By releasing inorganic ions, these Nps can effectively fill the enamel's micropores [12]. Due to their high solubility, quick ion release, ability to produce active oxygen species, and other properties, these nanosized particles also have good antibacterial effects [13]. These particles act similarly to the apatite crystals seen in biological enamel due to their shape and crystallinity. A recent study showed that enamel resistance to demineralization was enhanced through pH cycling by resin-based infiltrates doped with hydroxyapatite nanorods [14]. However certain metal ions like zinc and silver can be added to hydroxyapatite to enhance its antimicrobial effects. A property of copper is its potent antibacterial action. It has been demonstrated that exposure to copper surfaces, copper ions in solution, and suspensions of copper Nps will kill both gram-negative and gram-positive bacteria [15]. A study by Ghosh et al. investigated the antibacterial properties of copper-doped hydroxyapatite coatings against Staphylococcus aureus (*S. aureus*) and Escherichia coli (*E. coli*) [16]. The study concluded that due to the delayed release of copper ions from the coatings, the material demonstrated copper concentration-dependent antibacterial action against both types of bacteria. According to a study by Ravi et al., strontium-substituted hydroxyapatite showed a 56% and 35% decrease in microbial growth for *E. coli* and *S. aureus*, respectively [17]. The antimicrobial properties of HA Nps in orthodontics composites have been discussed previously [18]. However, the effect on the antimicrobial properties after the addition of metal ions like strontium and copper in orthodontic composite needs to be explored. The present study is the first to incorporate CuSrHA Nps in the orthodontic composite. The present study aimed to characterize and assess the antimicrobial properties of strontium and copper-doped HA Nps in an orthodontic composite Transbond XT^TM^ (Ormco Corporation, Glendora, USA).

## Materials and methods

The present in vitro study was conducted at Saveetha Dental College, Chennai. Institutional ethical clearance was obtained before the commencement of the study (IHEC/SDC/UG-1926/23//91).

Preparation of SrCuHA Nps

The preparation of strontium and copper co-substituted hydroxyapatite (SrCuHA) nanoparticles (Nps) was based on the techniques used by Salahuddin et al. [19] and Sodagar et al. [20]. First pure HA Nps was prepared using 0.1 M individual solutions of calcium nitrate tetrahydrate (Ca(NO_3_)_24_H_2_O) (Merck Group, Darmstadt, Germany) and 0.6 M ammonium dihydrogen phosphate (NH_4_H_2_PO_4_) (Merck Group, Darmstadt, Germany). The pH was adjusted to 11 using aqueous ammonia. To form pure HA Nps, the NH_4_H_2_PO_4_ solution was added dropwise to the Ca(NO_3_)_24_H_2_O solution and stirred vigorously for two hours. When a white-colored precipitate was formed, the solution was transferred to a Teflon-lined autoclave and subjected to hydrothermal treatment in a furnace at 200°C for five hours. The solution was then cooled to room temperature, filtered, washed several times with deionized water, and dried in an air oven at 100°C overnight. Powdered forms of pure HA Nps were obtained. To the pure HA Nps, Sr(NO_3_)_39_H_2_O (Merck Group, Darmstadt, Germany) and Cu(NO_3_)_2.6_H_2_O (Merck Group, Darmstadt, Germany) solutions were added dropwise, and the mixture was stirred for two hours. When the white-colored precipitate was formed, the solution was transferred to a Teflon-lined autoclave and subjected to hydrothermal treatment in a furnace at 200°C for five hours. The respective solution was then cooled to room temperature, filtered, washed several times with deionized water, and dried in an air oven at 100°C overnight. This produced powdered forms of SrCuHA Nps. This synthesized SrCuHA nanoparticle was characterized using Fourier transform infrared spectroscopy (FTIR), scanning electron microscopy (SEM), and energy-dispersive X-ray analysis (EDAX).

Characterization of SrCuHA Nps

The SrCuHA Nps were characterized using FTIR, for EDAX before integrating into the orthodontic composite. The chemical composition of the SrCuHA Nps was determined for EDAX. The functional group analysis of the Nps was investigated using FTIR in the frequency range of 4000-400 cm^-1^ (ALPHA 2, Bruker Corporation, Billerica, USA). The surface morphology of the SrCuHA Nps was studied using high-resolution scanning electron microscopy (HR-SEM) (JSM IT-800, JEOL, Ltd., Akishima, Japan).

Incorporation of the SrCuHA Nps into the orthodontic composite

The prepared SrCuHA Nps was incorporated into commercially available orthodontic composite Transbond XT^TM^ (Ormco Corporation, Glendora, USA). The incorporation technique used was based on the study by Reddy et al. [21]. About 200 mg of SrCuHA Nps were mixed with 2 g of orthodontic composite in a semi-dark environment using a glass spatula. The Nps mixed with composite was placed in a vortex mixer (Labquest, Borosil, India) with 600 rpm speed of rotation for 10 minutes. To prevent water dispersion into the prepared nanoparticle composite, it was placed in previously washed and capped beakers, and to avoid light exposure into the composite. The beakers were covered with black Teflon tape and then sonicated for 60-90 minutes. To maintain the stable temperature in the composite, ice cubes were added to water in the ultra-sonication system. The obtained SrCuHA Nps-incorporated composite was now kept in a dark airtight container for antimicrobial testing. The obtained nanocomposite was dissolved in the ratio of two different concentrations (20 μl and 40 μl) in 1 ml of dimethyl sulfoxide. Thus, one sample group containing a higher concentration of Nps (40 μl) and another sample containing a lower concentration of Nps (20 ml) were obtained. The obtained SrCuHA Nps-incorporated composite structure was analyzed using HR-SEM (JSM IT-800, JEOL, Ltd., Akishima, Japan).

Assessment of antibacterial property

The infection-causing gram-positive bacteria of *S. aureus* (MTCC-740), Streptococcus mutans (*S. mutans*) (MTCC-890), and gram-negative bacteria *E. coli* (MTCC-443) were maintained on a nutrient broth flask that contained all the essential nutrients to inhibit the growth of microbes. The test bacterial suspensions (20 μl) containing 150 cells ml^−1^ were used.

The agar well diffusion method was used to assess the antimicrobial efficacy. About 100 mL of Mueller Hinton agar for *S. mutans*, *S. aureus*, and *E. coli* were prepared, sterilized, and poured onto the Petri plates. The plates were allowed for solidification. After solidification, the respective plates were swabbed with the prepared bacterial suspensions. After swabbing, four wells on each plate were formed using a gel puncher. To those four wells, SrCuHA Nps-incorporated composite was loaded as two concentrations of 20 µL and 40 µL. Another well was filled with a control containing an antibiotic mixture of streptomycin and amoxicillin. The plates were then incubated at 37°C for 24 hours. Positive test results were scored when a zone of inhibition (ZOI) was observed around the well after the incubation period.

## Results

The characterization of the prepared SrCuHA Nps was done.

FTIR spectroscopy

**Figure 1:**
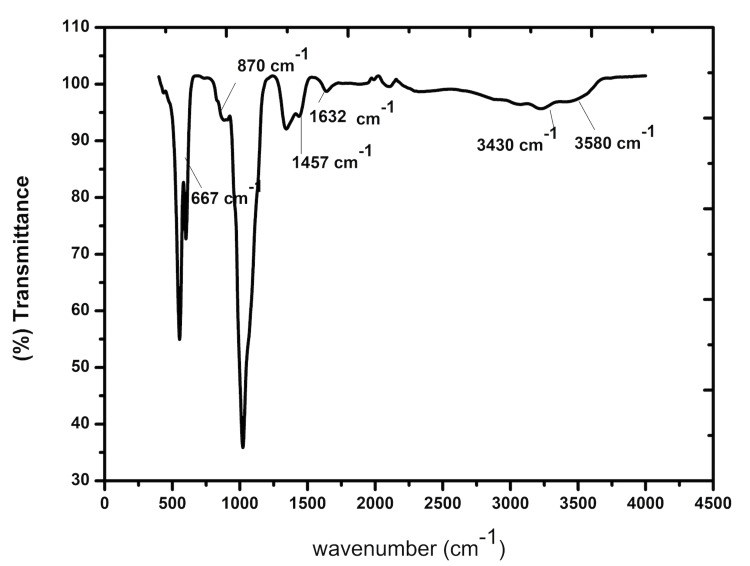
FTIR spectroscopy image of the SrCuHA Nps. Between 900 cm^-1^ and 1100 cm^-1^ and 550 cm^-1^ and 667 cm^-1^, respectively, are the characteristic absorption bands of the PO_3_^4-^ group. Due to the -OH group in SrCuHA, low-intensity absorption bands are present at 3580 cm^-1^ and 667 cm^-1^. H_2_O molecules are represented by the peaks at 3430 cm^-1^ and 1632 cm^-1^ in SrCuHA. FTIR: Fourier transform infrared spectroscopy; SrCuHA Nps: Strontium and copper co-substituted hydroxyapatite nanoparticles

Figure 1 displays the FTIR spectra for the CuSrHA Nps before integrating with the orthodontic composite. In the SrCuHA structure, the peak locations between 900 and 1100 cm^-1^ and 550 and 667 cm^-1^ are connected to PO_4_^3-^ and are regarded as a fingerprint zone of hydroxyapatite structure. The stretching and bending vibrations of the -OH groups in SrCuHA were connected to the peak positions at 3580 cm^-1^ and 667 cm^-1^. Peaks at 3430 cm^-1^ and 1632 cm^-1^, which represent the stretching and bending modes of H_2_O molecules in SrCuHA, respectively, were observed. In SrCuHA, the CO_3_^2-^ groups may be found in the frequency ranges of 870 cm^-1^ and 1457 cm^-1^.

EDAX spectroscopy

**Figure 2:**
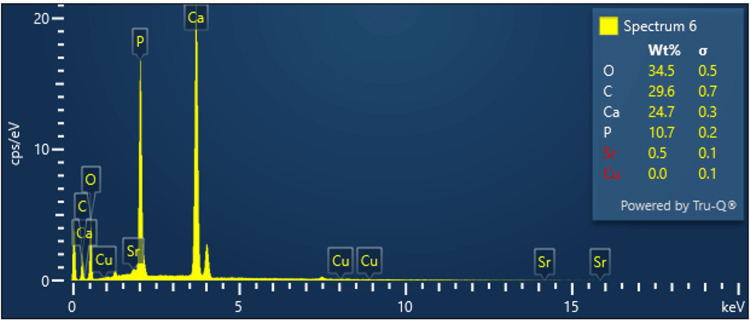
EDAX spectrum of the SrCuHA Nps. The peaks indicate the presence of dominant elements in the prepared SrCuHA nanoparticle. The dominant elements included Ca, P, O, C, Sr, and Cu. Cu: Copper; Sr: Strontium; C: Carbon; O: Oxygen; P: Phosphate; Ca: Calcium; EDAX: Energy-dispersive X-ray analysis; SrCuHA Nps: Strontium and copper co-substituted hydroxyapatite nanoparticles

The EDAX spectrum in Figure 2 shows that the dominating elements on the SrCuHA nanoparticle are Ca, P, Sr, Cu, and O, and thus the spectrum supports the formation of SrCuHA Nps. The mapping results provide evidence of a homogeneous distribution of mineral ions.

SEM analysis

**Figure 3:**
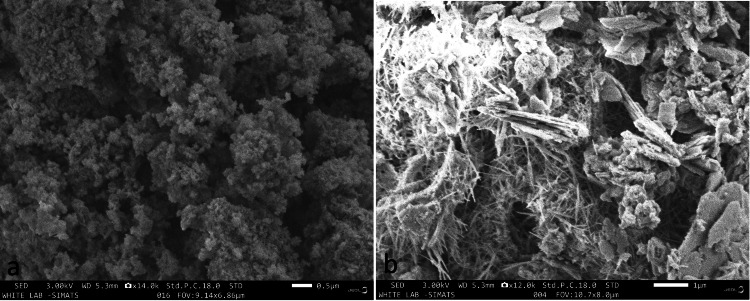
SEM image of the SrCuHA Nps and SrCuHA Nps integrated composite. a) SEM image of SrCuHA Nps, b) SEM image of the SrCuHA nanoparticles integrated into an orthodontic composite. SEM: Scanning electron microscopy; SrCuHA Nps: Strontium and copper co-substituted hydroxyapatite nanoparticles

HR-SEM was done to analyze the structure of SrCuHA Nps and also the SrCuHA-incorporated orthodontic composite. Figures 3a and 3b show the SEM image of the SrCuHA nanoparticle and the SrCuHA Nps integrated into the orthodontic composite, respectively. Figure 3a shows highly agglomerated Nps. The particles had a porous and unordered morphology. Figure 3b shows the SEM image of the SrCuHA Nps after successful integration with the orthodontic composite. Co-substitution increased the stability of hydroxyapatite and orthodontic composite.

Antimicrobial property

**Figure 4:**
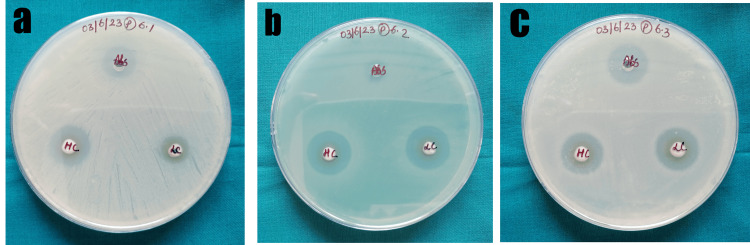
a) ZOI against S. mutans, b) ZOI against S. aureus, c) ZOI against E. coli. ZOI: Zone of inhibition; S. mutans: Streotococcus mutans; S. aureus: Staphylococcus aureus; E.coli: Escherichia coli

Table 1 and Figure 4 represent the measured ZOI for all the three groups. The maximum inhibition of *S. aureus* was seen in the control group (14 mm) followed by a 20 ml concentration of the SrCuHA Nps-incorporated composite (12 mm). With regard to *S. mutans*, the maximum ZOI was 40 ml of the SrCuHA Nps-incorporated composite (16 mm) followed closely by the control group (15 mm). The maximum ZOI of *E. coli* was by the control group (19 mm) followed by 40 ml of the SrCuHA Nps-incorporated composite (17 mm).

**Table 1:** Zones of inhibition of the three groups against microorganisms (S. aureus, S. mutans, and E. coli).

Group	Zone of inhibition		
	S. aureus	S. mutans	E. coli
Control group	14 mm	15 mm	19 mm
40 ml of SrCuHA Nps composite	10 mm	16 mm	17 mm
20 ml SrCuHA Nps composite	12 mm	11 mm	15 mm

## Discussion

In the current study, SrCuHA Nps were successfully integrated into the orthodontic composite. Utilizing FTIR, EDAX, and SEM imaging, the Nps were characterized. It verified the successful formation of the nanoparticle. SEM analysis of the SrCuHA Nps-incorporated composite was done. It verified the successful production and uniform inclusion of the Nps in the composite.

Korowash et al. characterized and investigated the antibacterial property of powdered strontium-copper-doped hydroxyapatite against *S. mutans *and *Candida albicans* using the well diffusion method [22]. The study reported ZOI against *S. mutans* that were similar to the present study. Huang et al. investigated the antibacterial capabilities of SrCuHA nanoparticle coating on implants [23]. It was found that the SrCuHA coating showed the maximum activity against the *E. coli* compared to the control group and pure hydroxyapatite. The antimicrobial results demonstrated (antimicrobial ratio 90.7%) that copper ions generated by SrCuHA have some bactericidal impact against *E. coli*. In the present study, the maximum ZOI was in the control group followed closely by high concentration of the SrCuHA Nps-incorporated composite. Another study investigated the antibacterial capabilities of strontium-copper co-doped mesoporous bioactive glass [24]. They concluded that as the nanoparticle ion content increased, so did the ZOI. The present study also found increased ZOI by the composite with a higher concentration of the SrCuHA Nps except against *S. aureus*.

Sodagar et al. studied the antimicrobial properties of silver hydroxyapatite nanocomposite against *S. mutans*, *Lactobacillus acidophilus*, and* Streptococcus sanguinis *[20]. They found that silver Nps had reduced the growth of cariogenic bacteria *S. mutans* but not the noncariogenic bacteria. The antibacterial properties of silver nanomaterials against a variety of microorganisms are well recognized [25]. Other metals, alloys, and metal oxides are known to have antibacterial properties and offer a potential avenue for treating antibiotic-resistant forms of bacteria while receiving much less research than silver [26]. The present study utilized copper and strontium to improve the antimicrobial efficacy of hydroxyapatite and showed comparable ZOI against *S. mutans* similar to the previous study. In a study, Argueta-Figueroa et al. investigated the antibacterial properties of composite materials containing copper Nps [27]. They discovered that the copper nanoparticle-containing composites significantly inhibited the growth of *S. aureus, E. coli*, and* S. mutans*. Yassaei et al. examined the antibacterial effects of orthodontic composites containing various Nps on *S. mutans* over time, including hydroxyapatite, titanium oxides, zinc oxides, copper oxides, and silver oxide Nps [28]. Short-term bactericidal effects are produced when 1% copper oxide and 1% silver oxide are added.

Limitations

The present study was a preliminary in vitro study and hence the results are not generalizable. The antibacterial property of the nanoparticle was assessed at one point in time only. The antimicrobial properties of the SrCuHA Nps-incorporated composite must be assessed in different time durations for its clinical application. Further extensive studies exploring the antimicrobial properties of SrCuHA Nps-incorporated composite against other oral pathogens, the effect of the nanoparticle on the bond strength, changes in mechanical properties, and the biocompatibility of the SrCuHA Nps doped composite must be evaluated for its clinical application.

## Conclusions

The CuSrHA Nps were synthesized and integrated into an orthodontic composite. The Nps were characterized using EDAX, FTIR, and SEM showing successful dispersion of the CuSrHA particles into the composite structure. The SrCuHA Nps-incorporated composite at 40 μl concentration showed the highest ZOI against *S. mutans* and comparable ZOI as the control group against *E coli*. SrCuHA Nps-incorporated composite at 20 μl showed the highest ZOI against *S. aureus*. The CuSrHA nanoparticle is a promising compound for incorporation into an orthodontic composite for its antimicrobial properties.
